# Volumetric Evaluation of Maxillary Lateral Incisor Root Resorption due to Positional Variations of Impacted Canine

**DOI:** 10.1155/2022/2626222

**Published:** 2022-06-09

**Authors:** Mohammed Hossein Razeghinejad, Roghieh Bardal, Saeid Shahi, Elham Mortezapoor, Maryam Mostafavi

**Affiliations:** ^1^Department of Orthodontics, School of Dentistry, Urmia University of Medical Sciences, Urmia, Iran; ^2^Department of Oral and Maxillofacial Radiology, Dental Caries Prevention Research Center, Qazvin University of Medical Sciences, Qazvin, Iran; ^3^Urmia University of Medical Sciences, Urmia, Iran; ^4^Department of Oral and Maxillofacial Radiology, School of Dentistry, Urmia University of Medical Sciences, Urmia, Iran

## Abstract

**Introduction:**

The purpose of this study was to investigate the relationship of the volume of lateral incisor resorption and impacted canine features.

**Materials and Methods:**

This study consisted of CBCT images of 47 samples with unilateral impacted maxillary canine (13 males and 34 females). The volume of lateral incisors in impacted side and nonimpacted side was calculated with the Mimics 10.01 software. Canine and lateral incisor angulations were measured in panoramic reformatted images. The canine cusp tip distance from midpalatal suture was measured in axial cross-section images.

**Results:**

The difference between two sides volume was considered as the mean volume of resorption (MVR) that was statistically significant (*P* < 0.001). MVR was not statistically significant between two sexes (*P*=0.95), in buccopalatal and mesiodistal positions of impacted canine, and in different angulations or distances of the impacted canine to midline (*P* > 0.05). The concurrent effect of the canine distance to the midline and the angle of the canine with the lateral incisor on the MVR were statistically significant (*P*=0.049).

**Conclusion:**

The maximum rate of lateral root resorption is when the distance from the canine to the midline is less than 5 mm and the angle of the canine to the lateral incisor is 30–60 degrees.

## 1. Introduction

Tooth impaction can be defined as failure in tooth eruption at the proper time and place in the dental arch during the normal developmental eruption [1–3].

Root resorption of teeth adjacent to impacted canine, especially the lateral incisor, is the most important and common complication that has irreparable consequences and can even cause loss of the adjacent teeth. Most root resorption occurs in the lateral incisor and then in the central incisor and rarely affects the premolars [3–5].

Superimposition of the lateral canine and lateral incisor, often seen in 2D radiographs, causes an error in the diagnosis of root resorption; on the other hand, root resorption of less than 0.6 mm in diameter and 0.3 mm in depth is undetectable in this type of radiography. Assessing root surface resorption requires 3D information [6].

Extent of root resorption of lateral incisors has been evaluated qualitatively in various studies, and effect of root morphology [7–10], type of inclusion of lateral incisor tooth, contact relationship between the canine and the lateral incisor, eruption inclination, and gender has been shown on this event. In the study of Ucar et al. [11], lateral incisor root resorption was assessed quantitatively, and the effect of several angular positions of impacted canine on the volume of lateral incisor root resorption was investigated; in the present study, the effect of the unilateral impacted canine linear and angular positions on the volume of the lateral incisor root resorption was investigated.

## 2. Methods and Materials

This retrospective study was performed using CBCT images of the maxilla with unilateral impacted canine that were collected from the archives of radiology offices located in northwest of Iran. Images were obtained for routine dental treatments. The sample size was determined 50, according to the mean and standard deviation of the lateral incisor volume of the impacted side (${\bar{X}}_{1}\pm SD_{1}$) and opposite side (${\bar{X}}_{2}\pm SD_{2}$) in Ucar et al.' study [11] and assuming a correlation coefficient of 0.5 between two groups:

(1) $$\begin{array}{c} n=\frac{{\left(z_{\alpha /2}+Z_{\beta }\right)}^{2}}{d^{2}},\\ d=\frac{\left({\bar{X}}_{1}-{\bar{X}}_{2}\right)}{SD\left({\bar{X}}_{1}-{\bar{X}}_{2}\right)},\\ \text{Power}=0.8,\\ \alpha =0.05. \end{array}$$

Images with existence and eruption of all permanent teeth except the third molar, with unilateral canine impaction, without physical syndromes, and history of maxillofacial trauma or history of orthodontic treatment were included in the study.

Three images with severe artifacts interfering outline detection of lateral incisor were excluded from the study.

**Figure 1:**
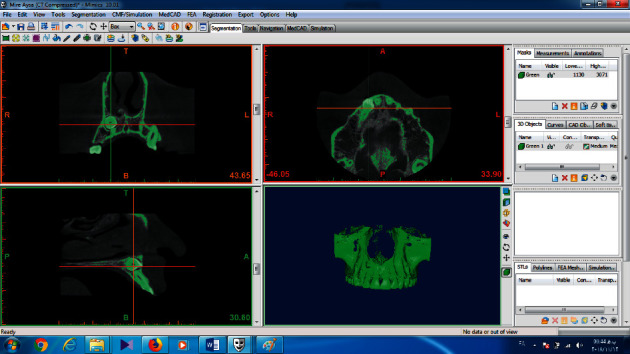
Lateral tooth and surrounding tissues isolated with mask.

**Figure 2:**
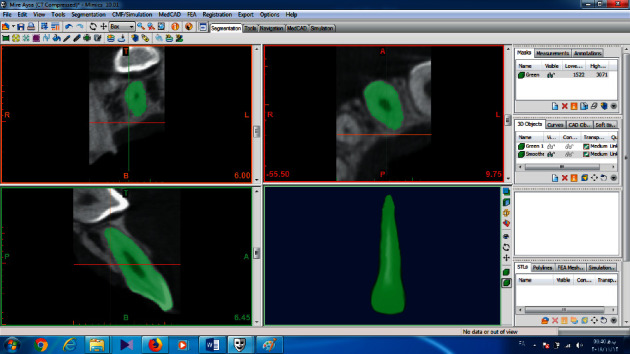
3D view of maxillary lateral of the impacted side.

**Figure 3:**
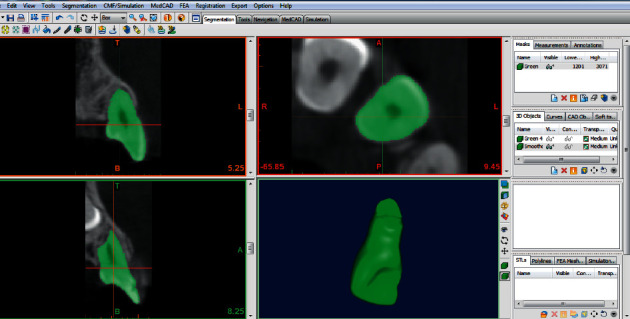
3D view of a severe resorption in lateral root CBCT.

Images were saved as DICOM files. All angular, linear, and volumetric measurements were done by an experienced maxillofacial radiologist, and all images were recorded on a 14-inch LCD screen (ASUS, China) with 1366 × 68 resolution in a room with moderate light level. In the Mimics 10.01 software, the upper, lower, mesial, and distal limits of lateral incisor were determined and separated with a mask from the surrounding tissues (Figure 1). Next, in all transverse slices with a 0.15 mm thick, areas of the image that did not belong to the teeth, such as PDL and surrounding bone, were removed, and in the sagittal and frontal sections, the limits of the mask were defined and reviewed. Finally, the 3D object was reconstructed, and the volume was measured in cubic millimeters by the software (Figures 2 and 3).

**Figure 4:**
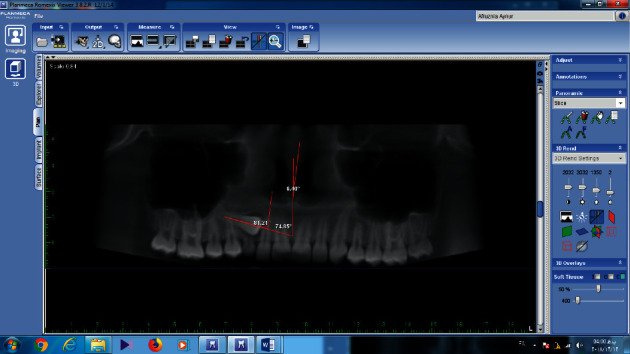
Measurement of canine angle with midline, canine angle with lateral, and lateral angle with midline on reformatted panoramic images of CBCT.

Linear and angular measurements were performed on panoramic images extracted from CBCT by the Romexis software (3.8.2); and longitudinal axis of the impacted canine, lateral incisor, and the midline were drawn, and the angle between the impacted canine and the lateral, the angle between the impacted canine and the midline, and the angle between the lateral and the midline were measured (Figure 4).

**Figure 5:**
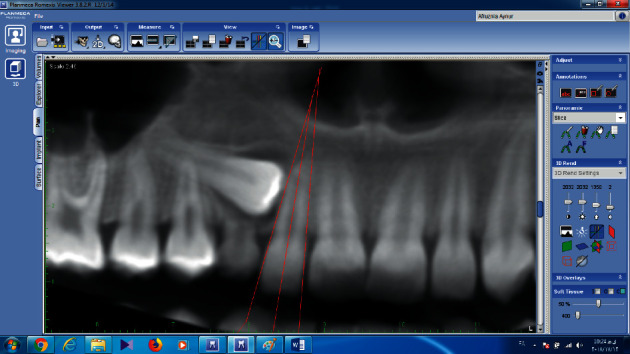
Longitudinal axis and marginal ridges for positioning with the Lindauer method.

Four positions were determined by the Lindauer method for determining the overlap between the impacted canine and lateral [12]; lines tangent to the mesial (a) and distal contours (b) as well as the line along the longitudinal axis of lateral incisor (c) was drawn. Position of P1 denotes that location of canine crown tip was distally to “b,” P2 was between “b” and “c,” P3 was between “a” and “c,” and P4 was more mesially than “a” (Figure 5).

**Figure 6:**
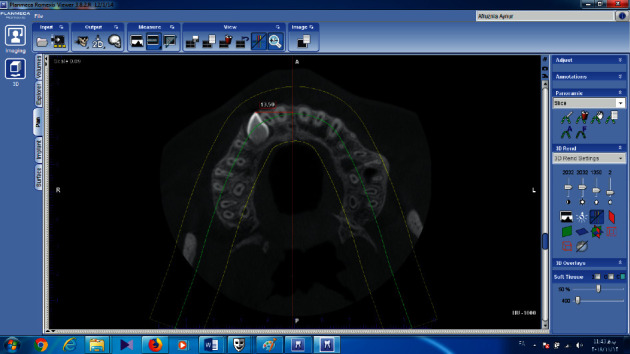
Measure the distance between the canine and midline on the axial sections.

The distance between canine and midline and canine crown tip with midpalatal suture was measured in the cross-sections (Figure 6).

The buccopalatal position of the canine was determined in the cross-sections as follows: the dental arch was drawn and the position of the canine crown relative to this line was determined.

### 2.1. Statistical Analysis

20% of the samples were observed by the second observer after two weeks, and the data were analyzed by ICC (intraclass correlation coefficients).

Statistical analysis was performed using the SPSS 20 software. The paired sample *t*-test was used to compare lateral incisor volume on the impaction side and contralateral side. One-way ANOVA was used to compare the volume of resorption based on mesiodistal position and the angle between the impacted canine with lateral and midline. Comparison of lateral incisor resorption in the two groups of male and female patients as well as in two groups of buccal and palatal impaction of canine and in the groups based on the distance between the canine and midline were performed by the independent *t* test.

Two-way ANOVA was used to compare the volume of resorption in terms of the simultaneous effects of canine to midline distance and lateral and canine angle. Pearson correlation coefficient was used to relation analyzes between angular and distance or volumetric measurements.

## 3. Results

The ICC index was calculated over 98%, which confirms the reliability of the measurements.

A total of 47 CBCT images of patients with unilateral maxillary canine impaction were examined.  Table 1 provides the descriptive information of these samples.

The mean lateral volume on the impacted side was 309.03 ± 68.02 mm^3^, this volume on the contralateral side was 336.57 ± 67.86 mm^3^. The difference between two sides volume was considered as MVR that was statistically significant (*P* < 0.001).

The MVR in men was 22.50 ± 20.14 and in women was 26.22 ± 20.64 mm^3^, and the difference between the two groups was not statistically significant (*P*=0.950).

The MVR in the buccal impaction was 25.56 ± 20.45 and in the palatal impaction was 21.53 ± 21.53 mm^3^, and there was no statistically significant difference between these locations (*P*=0.697).

There was no statistically significant difference in MVR based on mesiodistal position of the impacted canine (Table 2; *P*=0.480).

The mean angle of the impacted canine and lateral incisor was 53.81° ± 17.49° and there was no statistically significant relationship between this angle and MVR (Table 3; *P*=0.74).

The mean angle of the impacted canine with midline was 44.27° ± 16.53°. There was no significant association between this angle and MVR (Table 4; *P*=0.78).

The mean distance of impacted canine to midline was 6.19 ± 4.16 mm. There was no statistically significant relationship between this distance and MVR (Table 5; *P*=0.731).

The concurrent effect of the canine distance to the midline and the angle of the canine with the lateral incisor on the MVR was statistically significant (*P*=0.049; Table 6).

There was a inverse relation between the canine to midline distance and the angle between canine and lateral teeth (*P*=0.005; *R* = −0.40); and there was inverse relation between MVR and the angle between lateral and midline (*P*=0.05, *R* = −0.28) that was not statistically significant.

## 4. Discussion

The prevalence of maxillary canine impaction is between 1 and 3% [1]. Numerous factors can lead to impaction of the maxillary canine tooth. Local factors such as obstruction of the eruption pathway by lesions such as odontoma, deviation, or cessation of eruption due to the presence of chronic periapical granuloma of the deciduous canine, displacement of the unerupted permanent canine, and dilaceration of developing root due to trauma can lead to maxillary canine impaction [13]. Broadbent guidance theory of eruption of the maxillary anterior teeth states that the downward eruption of the canine dictates by the distal surface of the lateral incisor root [14]. Becker et al.' study showed that the prevalence of palatally impacted canines was significantly higher in people with missing, peg-shaped, or small lateral incisors [15]. The guidance theory of canine impaction states the role of genetic factors as determining environmental factors, including lateral tooth anomalies, and considers environmental factors as determining the eruption or impaction of the canine teeth [13]. Due to the different environmental conditions between the two sides of the maxilla, the prevalence of unilateral impaction is higher than bilateral cases. On the other hand, because the root apex, which determines the original location of the tooth germ, is determined by genetic factors, in cases where there is a mislocation of the apex, the canine impaction will be bilateral [16].

Impacted canine is associated with resorption of the adjacent lateral incisor. Early detection of canine impaction and root resorption of the maxillary lateral incisor can prevent many complications. Also, the diagnosis of root resorption and its severity can be effective in treatment planning [3]. Due to the presence of superimposition in 2D images, it is not possible to accurately detect root resorption, so CBCT images are required.

Previous studies on CBCT images have addressed root resorption qualitatively [4, 17, 18]. In this study, root resorption was quantified; also, some indices such as the impacted canine angle with lateral and midline as well as lateral angle with midline and canine and lateral overlap were considered on panoramic images reconstructed from CBCT to evaluate the predictability of maxillary lateral incisor resorption on panoramic images based on these indices.

In the present study, there was a significant relationship between canine impaction and lateral incisor root resorption, which agrees with the findings of Ucar et al. and Guarnieri et al. [4, 11].

The mean lateral volume in the present study was 309.03 mm^3^ on the impaction side and 336.57 mm^3^ on the contralateral side. In the study by Ucar et al., the mean lateral volume was 401.95 mm^3^ on the impaction side and 433.54 mm^3^ on the nonimpaction side [11]. Although using the same method for calculating the tooth volume in both studies, the lateral tooth volume in this study was lower than that of Ucar et al. This difference could be due to ethnicity, but in both studies, the lateral volume of the impaction side was less than that of the control.

No statistically significant differences were found in this study in terms of gender, which is similar to the results of studies by Ucar et al. and Cernochova et al. [5, 11], but studies by Cuminetti et al. [19], Chaushu et al. [20], and Ericson and Kurol [21] showed a higher prevalence of root resorption in women than in men. However, unlike the present study, these studies reported the resorption not quantitatively.

In the present study, 5 (10.6%) cases of impaction were in buccal position and 42 (89.4%) cases of impaction were observed in palatal position. In a literature review, Alqerban et al. found that different studies reported different figures on the prevalence of buccal and palatal impaction, and palatal impaction varied between 41% and 93% in studies [3]. This difference may be due to differences in sample size, mean age, race, and diagnostic methods.

The present study did not find a statistically significant relationship between buccal or palatal impaction and MVR, which is consistent with the study by Ucar et al. [11], but in Cernochova et al.' study, a relationship was observed between the impacted buccopalatal canine position and the intensity of resorption, with the largest amount of lateral incisor resorption being in the buccal position of the impacted canine [5]. In a study by da Silva Santos et al., lateral incisor resorption was more associated with palatal canine impaction [22].

In the present study, there was no statistically significant difference between the different positions of impacted canine divided into four groups by the Lindauer method, with respect to the mean volume of lateral maxillary incisor resorption. This finding is in line with the results of Ucar et al. [11]. Also, in the study by Guarnieri et al., there was no significant relationship between the canine and lateral overlap (using the Lindauer method) and degree and intensity of resorption [4].

In the present study, no statistically significant difference was observed between canine and midline angle or canine and lateral angle with MVR, which is similar to the results of the study by Ucar et al. [11]. In the study of Cernochova et al., where the longitudinal axis gradient of canine was divided into four groups of mesial, distal, vertical, and horizontal, no significant difference was observed between longitudinal axis gradient and lateral root canal resorption [5]. In Guarnieri et al.' study, there was a statistically significant association between canine and lateral angle and the volume of resorption, but the difference in resorption with respect to canine angle with midline and canine angle with occlusal plan was not significant [4].

In the present study, no significant relationship was found between canine and midline distance and the MVR, which is similar to the results of Ucar et al. [11]. Also, according to the results of the study by Cuminetti et al., with the decrease in canine to midline distance, the rate of cases with severe resorption was significantly higher, but the difference was not significant [19]. In the study of Farokh-Gisour et al., the mean distance of canine to midline in females was significantly lower than in males, which may be due to the small size of the maxilla in females, thus requiring further studies with larger sample sizes to separately examine male and female patients with respect to the relationship between canine and midline distance and lateral incisor resorption [1].

Since the effects of factors such as canine angle with midline, canine angle with lateral, canine to midline distance, and canine and lateral overlap on the resorption of the lateral tooth adjacent to the impacted canine were not significant alone, the effect of concurrency of these factors was investigated. The results showed that the volume of resorption was significantly associated with two factors of the canine to midline distance and the canine to the lateral angle. The highest volume of resorption is when the canine angle is 30°–60° and the canine to midline distance is less than 5 mm, while the least volume of resorption is related to the 30°–60° angle and distance of more than 5 mm. According to the results of the study by Guarnieri et al., lateral incisor resorption was significant in relation to the two factors of canine and lateral overlap and canine and lateral angle [4]. However, there is a need for further studies with larger statistical populations in this area.

In the present study, the relationship between the MVR and angle between lateral and midline was investigated. The results showed a significant negative correlation between the two variables, meaning that when the angle between the lateral and midline is low, further resorption is observed, and when this angle is high, that is, lateral is mesioangular and resorption is less. A large volume of resorption appears to occur in cases where the lateral tooth is not capable of mesioangulation against canine pressure; however, in some cases, the lateral angle with the midline was low, but no resorption had occurred. This was the case when the canine was located on the palatal side and at a distance from the lateral. Since this is the first attempt to investigate the relationship of lateral angle with the volume of resorption, further studies with larger sample sizes and considering the simultaneous effects of other factors are recommended.

## 5. Conclusion

The results of males and females were not statistically significant (*P*=0.950). There was no statistically significant difference between buccopalatal position (*P*=0.697). MVR was not statistically differed in different mesiodistal positions of impacted canine (*P*=0.480), different angulations between impacted canine and lateral incisor (*P*=0.74), different angulations of impacted canine with midline (*P*=0.78), and in different distances of impacted canine to midline (*P*=0.78).

The concurrent effect of the canine distance to the midline and the angle of the canine with the lateral incisor on the MVR were statistically significant (*P*=0.049). There was inverse relation between the canine to midline distance and the angle between canine and lateral incisor (*P*=0.005; *R* = −0.40).

The maximum rate of lateral root resorption is when the distance from the canine to the midline is less than 5 mm and the angle of the canine to the lateral incisor is 30–60 degrees.

## Figures and Tables

**Table 1:** Descriptive information of impacted canines.

	Male	Female	Left	Right	Buccal	Palatal	P_1_	P_2_	P_3_	P_4_
Number (%)	13 (27.7)	34 (72.3)	19 (40.4)	28 (59.6)	5 (10.6)	42 (89.4)	59 (10.6)	5 (10.6)	7 (14.9)	30 (63.8)

**Table 2:** Comparison of the MVR in terms of canine position in the mesiodistal dimension.

Position	Volume of root resorption (mm^3^)			*P* value
	*n*	Mean	SD	
P1	5	31.46	27.83	0.480
P2	5	13.00	9.11	
P3	7	26.75	17.26	
P4	30	25.19	21.00	

One-way ANOVA, *n*, number; SD, standard deviation.

**Table 3:** Comparison of the MVR in terms of the angle between impacted canine and lateral incisor.

Angulation	Volume of root resorption (mm^3^)			*P* value
	*n*	Mean	SD	
0°–30°	5	31.03	21.99	0.74
30°–60°	25	25.50	23.88	
60°–90°	17	23.03	14.11	

One-way ANOVA, *n*, number; SD, standard deviation.

**Table 4:** Comparison of the MVR in terms of the angle between impacted canine and midline.

Angulation	Volume of root resorption (mm^3^)			*P* value
	*n*	Mean	SD	
0°–30°	12	22.33	19.88	0.78
30°–60°	29	26.84	22.29	
60°–90°	6	22.93	10.86	

One-way ANOVA, *n* number; SD, standard deviation.

**Table 5:** Comparison of the MVR by distance between canine and midline.

Distance	Volume of root resorption (mm^3^)			*P* value
	*n*	Mean	SD	
<5 mm	25	27.05	21.27	0.731
>5 mm	22	23.08	19.54	

Independent *t*-test, *n*, number; SD, standard deviation.

**Table 6:** Comparison of the MVR in terms of simultaneous effects of the canine to midline distance and angle between the canine and lateral.

Angulation	Distance (mm)	Volume of root resorption (mm^3^)			*P* value
		*n*	Mean	SD	
0°–30°	<5	0	—	—	
	>5	5	31.73	21.99	
30°–60°	<5	13	33.26	25.68	0.049
	>5	12	17.10	19.41	
60°–90°	<5	12	20.33	13.13	
	>5	5	29.48	15.74	

Two-way ANOVA, *n*, number; SD, standard deviation.

## Data Availability

The data used to support the findings of this study are included within the article.

## References

[1] Farokh-Gisour E., Salahi-Ardakani M.-A., Motaghi R. (2016). Localization of impacted maxillary canines and root resorption of neighbouring latral incisor using cone beam computed tomography. *International Journal of Medical Research & Health Sciences*.

[2] D Oleo-Aracena M. F., Arriola-Guillén L. E., Rodríguez-Cárdenas Y. A., Ruíz-Mora G. A. (2017). Skeletal and dentoalveolar bilateral dimensions in unilateral palatally impacted canine using cone beam computed tomography. *Prog Orthod*.

[3] Alqerban A., Jacobs R., Lambrechts P., Loozen G., Willems G. (2009). Root resorption of the maxillary lateral incisor caused by impacted canine: a literature review. *Clinical Oral Investigations*.

[4] Guarnieri R., Cavallini C., Vernucci R., Vichi M., Leonardi R., Barbato E. (2016). Impacted maxillary canines and root resorption of adjacent teeth: a retrospective observational study. *Medicina Oral Patología Oral y Cirugia Bucal*.

[5] Cernochova P., Krupa P., Izakovicova-Holla L. (2011). Root resorption associated with ectopically erupting maxillary permanent canines: a computed tomography study. *The European Journal of Orthodontics*.

[6] Ericson S., Kurol J. (1988). CT diagnosis of ectopically erupting maxillary canines-a case report. *The European Journal of Orthodontics*.

[7] Brin I., Becker A., Zilberman Y. (1993). Resorbed lateral incisors adjacent to impacted canines have normal crown size. *American Journal of Orthodontics and Dentofacial Orthopedics*.

[8] Kook Y. A., Park S., Sameshima G. T. (2003). Peg-shaped and small lateral incisors not at higher risk for root resorption. *American Journal of Orthodontics and Dentofacial Orthopedics*.

[9] Sameshima G. T., Sinclair P. M. (2001). Predicting and preventing root resorption: Part I. Diagnostic factors. *American Journal of Orthodontics and Dentofacial Orthopedics*.

[10] Nigul K., Jagomagi T. (2006). Factors related to apical root resorption of maxillary incisors in orthodontic patients. *Stomatologija*.

[11] Ucar F. I., Celebi A. A., Tan E., Topcuoğlu T., Sekerci A. E. (2017). Effects of impacted maxillary canines on root resorption of lateral incisors: a cone beam computed tomography study. *J Orofac Orthop*.

[12] Lindauer S. J., Rubenstein L. K., Hang W. M., Andersen W. C., Isaacson R. J. (1992). Canine impaction identified early with panoramic radiographs. *The Journal of the American Dental Association*.

[13] Becker A., Chaushu S. (2015). Etiology of maxillary canine impaction: a review. *American Journal of Orthodontics and Dentofacial Orthopedics*.

[14] Broadbent B. H. (1941). Ontogenic development of occlusion. *The Angle Orthodontist*.

[15] Becker A., Smith P., Behar R. (1981). The incidence of anomalous maxillary lateral incisors in relation to palatally-displaced cuspids. *The Angle Orthodontist*.

[16] Ericson S., Kurol J. (1988). Early treatment of palatally erupting maxillary canines by extraction of the primary canines. *The European Journal of Orthodontics*.

[17] Ericson S., Kurol J. (2000). Incisor root resorptions due to ectopic maxillary canines imaged by computerized tomography: a comparative study in extracted teeth. *Angle Orthod*.

[18] Lai C. S., Bornstein M. M., Mock L., Heuberger B. M., Dietrich T., Katsaros C. (2013). Impacted maxillary canines and root resorptions of neighbouring teeth: a radiographic analysis using cone-beam computed tomography. *The European Journal of Orthodontics*.

[19] Cuminetti F., Boutin F., Frapier L. (2017). Predictive factors for resorption of teeth adjacent to impacted maxillary canines. *International Orthodontics*.

[20] Chaushu S., Kaczor-Urbanowicz K., Zadurska M., Becker A. (2015). Predisposing factors for severe incisor root resorption associated with impacted maxillary canines. *American Journal of Orthodontics and Dentofacial Orthopedics*.

[21] Ericson S., Kurol J. (1987). Incisor resorption caused by maxillary cuspids. a radiographic study. *Angle Orthod*.

[22] da Silva Santos L. M., Bastos L. C., Oliveira-Santos C., da Silva S. J. A., Neves F. S., Campos P. S. F. (2014). Cone-beam computed tomography findings of impacted upper canines. *Imaging Science in Dentistry*.

